# Editorial: Designing Bio-Formulations Based on Organic Amendments, Beneficial Microbes and Their Metabolites

**DOI:** 10.3389/fmicb.2021.832149

**Published:** 2022-01-18

**Authors:** Roberta Marra, Santiago Gutiérrez, Sheridan Lois Woo, Giuliano Bonanomi, Francesco Vinale

**Affiliations:** ^1^Department of Agricultural Sciences, University of Naples Federico II, Naples, Italy; ^2^Center for Studies on Bioinspired Agro-Environmental Technology (BAT Center), University of Naples Federico II, Naples, Italy; ^3^Area of Microbiology, University of León, Ponferrada, Spain; ^4^Department of Pharmacy, University of Naples Federico II, Naples, Italy; ^5^Task Force on Microbiome Studies, University of Naples Federico II, Naples, Italy; ^6^Institute for Sustainable Plant Protection, National Research Council, Naples, Italy; ^7^Department of Veterinary Medicine and Animal Productions, University of Naples Federico II, Naples, Italy

**Keywords:** microbial community, microbiomes, plant protection products, plant-microbe interactions, volatilome, proteome, metabolome, transcriptome

The future scenario in the agricultural sector, challenged by regulatory pressure, public concern and environmental issues, continues to motivate the development of alternative methods to chemicals for applications as fertilizers and pesticides (Martin, 2003). Among these, the use of organic amendments and microbial biocontrol agents represents the most promising soil management strategy, that may contribute both directly and indirectly to crop production and plant health (Bargaz et al., 2018).

The aim of this Research Topic was to provide insight in recent advances and challenges of plant-microbiomes studies focused on novel bio-formulations useful for the development of sustainable and eco-efficient approaches for plant disease control. The Topic includes 19 original research and review articles, which have been grouped in four categories.

## Modulation of Plant Response by Beneficial Microbes

The interactions between plant and microbes have attracted great attention in the last decades. Biotechnological applications of beneficial microbes (BMs) in different areas, e.g., stimulation of plant-growth, biocontrol, bioremediation of contaminated sites and production of bioactive compounds with pharmaceutical and industrial relevance have been evaluated (Wu et al., 2009). Several studies have investigated the molecular changes occurring in the host plant and the interacting microbe aiming to improve our understanding about how plants respond to microbial colonization or attack and how microbes affect plant cellular processes (Cheng et al., 2019; Adeniji et al., 2020; Jain et al., 2021). In their work, Bonini et al. showed that the metabolic profile in pepper leaves was modulated in response to microbial treatments consisting of arbuscular mycorrhizal fungi (AMF: *Rhizoglomus irregularis* and *Funneliformis mosseae*), and *Trichoderma koningii*. Root colonization by BMs increased total fruit yield, modified gibberellin, auxin, and cytokinin plant hormone patterns, as well as other secondary metabolism processes, determining the accumulation of carotenoids, saponins, and phenolic compounds. Similarly, Lombardi et al. found that selected *Trichoderma* strains stimulated the growth of strawberry plants and improved crop yield. Moreover, proteomic analysis of the produced fruits revealed a complex reprogramming of physiological processes e.g., stress response, nutrient uptake, protein metabolism, etc., that improved the health properties.

The application of microbial (e.g., AMF, *Trichoderma koningii* and rhizobacteria) or non-microbial (e.g., a protein hydrolyzate) biostimulants elicited biomass increase in maize plants and affected the metabolomic response in leaves and roots (Rouphael et al.). Interestingly, most of the differential metabolites consisting mainly of phenylpropanoids and terpenes were accumulated more in plant tissues following the application of the protein hydrolyzate in comparison to the treatments with microorganisms alone. The treatments also induced a reprogramming of the entire phytohormone profile, particularly in the roots, thus supporting the hypothesis that some biostimulants may have a hormone-like activity.

Conventional farming relies highly on N-fertilizers to improve crop yield, but this practice can lead to severe environmental pollution, hence the urgent need to find sustainable alternatives to inorganic fertilizers. Rubio et al. analyzed the transcriptomic response of wheat seedling roots inoculated with *Trichoderma harzianum* under different inorganic N supplies using wheat genome 61K microarrays. Forty-eight hours after inoculation, *Trichoderma* induced a greater expression of defense-related genes than the calcium nitrate application, that also down-regulated expression of genes involved in plant growth and development processes. Moreover, genes involved in the flavonoid biosynthetic pathway were differentially expressed in treated plants, thus reinforcing the hypothesis that *Trichoderma* is capable of inducing plant defense through the modulation of secondary metabolism.

## Effects of Microbial Metabolites on Plant-Microbe Interactions

Greater understanding about the effects induced by microbial metabolites on the host plant or by the host plant on the metabolism of the associated microbes could ultimately lead to the development of new crop protection bio-formulations for improving crop quality and yields. In their review, Tilocca et al. discussed the potential exploitation of volatile organic compounds (VOCs) produced by bacterial and fungal biocontrol agents (BCAs) in the control of many important phytopathogens. The authors underline the importance of the ecosystem in shaping the microbial VOCs composition. The adoption of holistic approaches, such as -omics and bioinformatics prediction tools, may help scientists to unravel the dynamic network of molecular cross-talk occurring between microbial partners and their plant hosts.

Members of the genus *Bacillus* are examples of well-known producers of antifungal compounds (Chaurasia et al., 2005). Zhang et al. analyzed the antifungal effects of twenty-nine VOCs produced by a novel strain of *B. subtilis* (ZD01) against the airborne plant pathogen *Alternaria solani*. VOCs produced by ZD01 caused a strong decrease in hyphae penetration, inhibition of conidia germination, and reduced virulence of the fungus on potato leaves. Moreover, the expression of two virulence-associated genes (*sod* and *slt2*) in *A. solani* was strongly down-regulated after exposure to ZD01 VOCs.

Marine-derived fungi are considered good producers of valuable compounds with original structures and interesting biochemical properties that can be exploited for new drug discovery (Silber et al., 2016; Nicoletti and Vinale, 2018; Vinale et al., 2020). Zhao et al. isolated two novel fusarisetins, namely fusarisetins C and D, and four known compounds produced by the fungus *Fusarium equiseti* D39. These 3-decalinoyltetramic acid (3DTA) derivatives showed potent phytotoxicological and antimicrobial activities. Moreover, the optimization of fermentation conditions favoring equisetin production was conducted by using the “one strain many compounds” (OSMAC) approach.

A new method to rhizosphere engineering proposes the inoculation of microbial consortia to emulate the complex biological networks in natural soils, thus recruiting beneficial microbes and establishing optimized plant microbiomes (Syed Ab Rahman et al., 2018; Woo and Pepe, 2018). Inoculation with endophytes was found to stimulate the biosynthesis of secondary metabolites (SMs) in medicinal plants (Pandey et al., 2016a,b, 2018). The inoculation of an endopyhytic-consortium, consisting of *Acinetobacter* sp. and *Marmoricola* sp., significantly increased the biosynthesis of benzylisoquinoline alkaloids including morphine and thebaine in poppy plants, as well as improvement in plant growth and yield (Ray et al.). Interestingly, the increment in metabolite content was related to the modulation of the biosynthetic pathway due to the complementary activities of the consortium member endophytes. Lin et al. observed that the production of plant alkaloids by the association between the fungal endophyte *Epichloë* and the perennial grass *Festuca sinensis* depended mainly on the environmental growth conditions. In field and greenhouse tests, the seasonal variation more than the plant ecotype significantly affected the concentrations of alkaloids (peramine, lolitrem B, and ergot) produced by *F. sinensis*.

Plant–microbe interactions can be exploited to enhance the production of important SMs having multiple roles (Singh et al., 2017). As an alternative approach to the application of phytohormones and plant growth regulators, Luziatelli et al. investigated the biostimulant properties of compounds secreted by *Pantoea agglomerans* strain C1, known to produce indole-3-acetic acid (IAA) and siderophores (Luziatelli et al., 2019). The *P. agglomerans* C1 metabolites effectively stimulated root formation and plant development of woody fruit crops, thus offering novel opportunities in designing innovative biostimulants.

Microbial metabolites have been proposed as alternatives to the use of living BCAs, because the efficacy of the latter is often limited by their low persistence in the environment, inconsistent performance under diverse field conditions and slower mode of action against pathogens, in comparison to chemical counterparts (Zaidi and Singh, 2013). Berini et al. investigated the activity of two metagenome-sourced microbial chitinases (Chi18H8 and 53D1) as potential insecticidal proteins against *Bombyx mori*, a model system among Lepidoptera. Although both proteins gave similar results *in vitro*, only 53D1 orally administered to *B. mori* larvae induced mortality and dramatically affected insect development. This study confirms that novel insecticidal formulations based on microbial bioactive proteins may be used in the progressive reduction of synthesized chemical compound applications, thus reducing the environmental impact of single active substances and the risk of resistance selection (Chandler et al., 2011; Hardy, 2014).

New microbial species or antimicrobial metabolites can be found by exploring environments having a high microbial biodiversity. Xu et al. observed that among insect symbionts, fungi isolated from the termite species *Odontotermes formosanus* demonstrated a great diversity and revealed the association with different bacterial symbionts. Moreover, the fungal metabolites exhibited antimicrobial activities when used in combination both as crude extracts and when applied as purified compounds. These results demonstrated that insect symbionts may represent a powerful source of novel species useful in agriculture or for drug discovery.

## Novel Bioformulations for Applications in Agriculture

*Trichoderma* spp. are well-known examples of BCAs used as active ingredients of many plant protection products for agriculture and are also known for their ability to promote plant growth and/or induce disease resistance (Woo et al., 2014; Woo and Pepe, 2018). Besides the continuous search for novel effective natural strains, numerous efforts have been carried out to obtain modified microbial isolates with improved abilities compared to wild type strains, produced by using processes including UV radiation or chemical mutagenesis (Papavizas et al., 1982). Mukherjee et al. developed a novel seed dressing formulation based on a *T. virens* mutant (named G2) obtained by gamma-ray-induced mutagenesis. In comparison to the wild-type strain, *T. virens* G2 revealed a superior ability to contrast pathogens, both *in vitro* and in greenhouse or field trials. Transcriptome analysis revealed an up-regulation of genes involved in the synthesis of SMs. The proposed formulation consisted of the *T. virens* G2, tamarind seeds as a fermentation medium and talcum powder as a carrier and demonstrated a better efficacy in controlling *Sclerotium rolfsii* in chickpea and lentil production. In addition, it exerted multiple beneficial effects on plant, but it did not have toxic effects on mammals, birds or fish. Commercial formulations of *Trichoderma* spp. have been also tested as systemic resistance inducers of plants attacked by the parasitic nematode *Meloidogyne incognita*. Pocurull et al., in agreement with previous studies (de Medeiros et al., 2017; Martínez-Medina et al., 2017), showed that *T. asperellum* T34 and *T. harzianum* T22 induced resistance in tomato plants against *M. incognita*. Interestingly, the resistance was also induced in tomato bearing the *Mi-1.2* root-knot nematode resistance gene, and this protective effect was additive to that observed in T34-inoculated plants. In the review by Poveda et al., *Trichoderma*, mycorrhizal and endophytic fungi were indicated as the main filamentous fungi used as BCAs or resistance inducers against plant-parasitic nematodes. Different mechanisms of action were discussed that were differentiated according to each group of fungi, including those employing the production of SMs and lytic enzymes, the competition for space and nutrients, or direct parasitism; and those relying on the activation of plant defense mechanisms, such as the activation of SAR and ISR, modification of root exudates composition, or production of strigolactones. The wide diversity of mechanisms used by this consortium of microorganisms may represent a valid durable strategy for the control of plant-parasitic nematodes, but several issues (e.g., the influence of local environment and the results in field experiments) require further investigations before BCA applications may be used effectively in nematode-control of a particular crop.

Romano et al. isolated and characterized plant growth-promoting bacteria (PGPB) from the rhizosphere of wheat plants cultivated under abiotic stresses as plant probiotics. Two isolates of *Kosakonia pseudosacchari* TL8 and TL13 were identified that showed multiple plant growth promotion activities as well as antimicrobial activity and stress tolerance. TL13, the best PGP performer, was selected to develop a novel microbial-based formulation obtained using agro-industrial by-products as carbon source. This permits a reduction of costs and the development of an eco-sustainable inoculant, resulting in an eco-friendly, easy and economically advantageous approach to manage by-products.

## Ecology and Functionality of Plant-Soil Microbial Community

Complex interactions are established in the soil microbial community with plant roots and soil components, that significantly influence plant growth and resistance toward multiple stresses (Vishwakarma et al., 2020). Understanding the processes in the rhizosphere, as well as unraveling the composition and biological functions of the plant-root microbiome becomes essential, since recent evidence has confirmed that host plants and their associated microbes function as metaorganisms or holobionts (Hacquard, 2016).

Rhizosphere microbial community structures is continuously adapting to the environment, particularly to the agronomic practices. Bacterial and fungal community structures were strongly affected by soil and soilless cultivation systems of tomato plants subjected to different fertilization strategies (Grunert et al.). In both cultivation systems, similar plant performance was observed, but multivariate analysis showed that physicochemical characteristics (e.g., plant length, pH, phosphorous, ammonium, potassium, etc.) changed and altered mainly the bacterial community composition. These results may offer novel opportunities for designing tailored fertilizers applicable to sustainable agriculture.

The development of novel bio-formulations also requires an in-depth study of the molecular factors regulating plant-microbe interactions and to possibly unravel the interactive mechanisms occurring in the rhizosphere (Vishwakarma et al., 2020). In the study by Pachauri et al. the characterization of the mutant M7 of *Trichoderma virens*, previously obtained by gamma-ray induced mutagenesis, showing morphological and metabolic deficiencies compared to the wild-type strain (Mukherjee et al., 2006), was completed. Transcriptome analysis showed that several genes involved in secondary metabolism, carbohydrate metabolism, hydrophobicity, and transportation were down-regulated in M7. In addition, a genetic deletion comprising a total of 250 kb, including 71 predicted ORFs, was found by whole genome sequencing. This approach could help in the identification of novel regulators resulting in the beneficial traits of *Trichoderma* fungi.

Despite the importance of plant-fungus interactions, the molecular factors involved in the regulation of these systems are not fully understood. By using the filamentous fungus *Neurospora crassa* as a model organism to study plant-fungal relationships, Martins et al. demonstrated that the deletion of the gene encoding the zinc finger transcription factor PAC-3, regulated by ambient pH, affected numerous physiological functions, including adaptation to nutritional conditions, regulation of virulence, or regulating the expression of genes associated with structural and metabolic features. These findings highlighted the pivotal role of PAC-3 as regulatory mediator involved in fungal pathogenesis.

## Conclusion

Currently, the most active research field of investigating integrated pest management strategies is the development of sustainable and eco-efficient approaches for plant disease control. The commercial development of a microbial formulation requires several steps including: isolation, identification and characterization of selected microbes and their biological activity; the optimization of fermentation processes, storage conditions, and the development of the formulation; the registration through opportune patenting, and commercialization (Montesinos, 2003). The articles included in this Research Topic cover different aspects of the interactions occurring in agroecosystems between plants and microbes that will facilitate the implementation of novel sustainable strategies for plant defense.

## Author Contributions

The authors defined the subject of this Research Topic and joined in the editing procedure. RM wrote the first draft of the manuscript. SW, GB, FV, and SG revised and improved the manuscript. All authors approved this editorial for publication.

## Funding

This work was supported by MISE CRESO (Grant number **PROTECTION** n. F/050421/01-03/X32); PRIN 2017 (Grant number **PROSPECT** 2017JLN833); MISE Sportello Agrifood DM 5/3/2018 (Grant **VIABIO**); European Union Horizon 2020 Research and Innovation Program, **ECOSTACK** (Grant agreement number 773554); MUR, PNR 2015-2020. ARS01_00985 **BIOFEEDSTOCK** - Sviluppo di Piattaforme Tecnologiche Integrate per la Valorizzazione di Biomasse Residuali; PSR Veneto 16.1.1 (Grant number **DIVINE** n. 3589659); PSR Campania 2014/2020 Misura 16.1 Azione 2 Progetto **DI.O.N.IS.O**. (C.U.P. B98H19005010009).

## Conflict of Interest

The authors declare that the research was conducted in the absence of any commercial or financial relationships that could be construed as a potential conflict of interest.

## Publisher's Note

All claims expressed in this article are solely those of the authors and do not necessarily represent those of their affiliated organizations, or those of the publisher, the editors and the reviewers. Any product that may be evaluated in this article, or claim that may be made by its manufacturer, is not guaranteed or endorsed by the publisher.
